# Severe Mental Illness Longitudinal Evaluation (SMILE): protocol for establishing a cohort and bioresource for UK-based patients with psychosis

**DOI:** 10.1136/bmjopen-2025-116077

**Published:** 2026-07-01

**Authors:** Anthony Quinn, Thomas Johnson, Julie Bieles, Lesley Booth, Robert A McCutcheon, John Bradley, Nathalie Kingston, Gerome Breen, Amelia Harshfield, Graham K Murray, James T R Walters, Benjamin I Perry, Mohammad Zia Ul Haq Katshu, Kimberley Kendall, Rachel Upthegrove

**Affiliations:** 1 Department of Psychiatry, University of Oxford, Oxford, Oxfordshire, UK; 2 TUNE-UP Service, Oxford Health NHS Foundation Trust, Oxford, UK; 3 Department of Psychosis Studies, Institute of Psychiatry, King’s College London, London, UK; 4 NIHR Cambridge Biomedical Research Centre, Cambridge Biomedical Campus, Cambridge, UK; 5 NIHR BioResource, Cambridge University Hospitals NHS Foundation Trust, Cambridge Biomedical Campus, Cambridge, UK; 6 Department of Medicine, School of Clinical Medicine, University of Cambridge, Cambridge Biomedical Campus, Cambridge, UK; 7 Addenbrookes Hospital, Cambridge University Hospitals NHS Foundation Trust, Cambridge Biomedical Campus, Cambridge, UK; 8 Department of Renal Medicine, Addenbrookes Hospital, Cambridge University Hospitals NHS Foundation Trust, Cambridge, UK; 9 Department of Haematology, School of Clinical Medicine, University of Cambridge, Cambridge Biomedical Campus, Cambridge, UK; 10 Social, Genetic and Developmental Psychiatry Centre, King’s College London, London, UK; 11 Department of Psychiatry, University of Cambridge, Cambridge, Cambridgeshire, UK; 12 Centre for Neuropsychiatric Genetics and Genomics, Division of Psychological Medicine and Clinical Neurosciences, School of Medicine, Cardiff University, Cardiff, UK; 13 Institute for Mental Health, University of Birmingham, Birmingham, UK; 14 Institute of Mental Health, University of Nottingham, Nottingham, Nottinghamshire, UK; 15 Nottinghamshire Healthcare NHS Foundation Trust, Nottingham, UK; 16 NIHR Oxford Health Biomedical Research Centre, Oxford, UK

**Keywords:** MENTAL HEALTH, Schizophrenia & psychotic disorders, Adult psychiatry, Genomic Medicine

## Abstract

**Abstract:**

**Introduction:**

Psychotic disorders account for significant morbidity and healthcare costs and yet their pathophysiology remains poorly understood. The National Institute for Health and Care Research (NIHR) Severe Mental Illness Longitudinal Evaluation (SMILE) BioResource is a collaborative project that aims to collect clinical data and biological samples from people with psychosis for long-term storage, future projects and recontact for targeted trials.

**Methods and analysis:**

The NIHR SMILE BioResource cohort will initially include up to 2000 UK-based patients with a recorded diagnosis of psychosis. Clinical symptoms will be captured using self-report and clinician ratings. Biosamples will enable genotyping and wider omics as further funding allows. Study data will be analysed to facilitate development of discovery science for underlying mechanisms of psychotic disorders and recall of participants for targeted interventional studies.

**Ethics and dissemination:**

This study is sponsored by the University of Oxford and received full ethical approval from Wales REC 2. SMILE BioResource biosamples and data will be stored long-term by the NIHR BioResource. Researchers who are interested in applying to use these biosamples and data, and/or recontacting SMILE participants can find further information on the website of the NIHR BioResource.

**STRENGTHS AND LIMITATIONS OF THIS STUDY**

- A multifaceted understanding of mental health, for example, biological, cognitive and sociodemographic factors.
- Provides possibilities for co-consent to research studies with a similar design.
- Currently only recruits participants who have capacity to consent, which may lead to selection bias.
- Challenges ensuring people from all sections of society, irrespective of ethnic or socio-economic background, are represented in this resource.^23^

## Introduction

 Psychotic disorders, including schizophrenia, schizoaffective disorder and affective psychosis, impact up to 3% of the population and can severely affect quality of life for individuals and their families, including the ability to function socially and professionally. People with psychotic disorders also die around 15 years earlier than the general population, largely due to cardiometabolic disease.^1 2^ Despite current best treatments, psychotic disorders represent a significant and unaddressed need. Individuals living with psychotic disorders represent a patient group whose illness has not received the research investment or potential equal to its personal, family and societal burden.

There is considerable diagnostic heterogeneity in psychotic disorders; herein, we use the terms severe mental illness (SMI) and ‘psychosis’ to encompass schizophrenia, schizoaffective disorder, affective and broader defined primary psychotic disorders, as defined by International Classification of Diseases (ICD)-11 (see Methods and analysis section).

Studies have reported different pathological factors that contribute to psychosis including genetic risk, subcortical dopamine and glutamatergic dysfunction, structural and functional grey and white matter changes, and cognitive impairment impacting executive function, attention and working memory.^3^,^5^ This better understanding of pathological processes may eventually lead to more specific and effective therapies, yet progress is slow. While considerable efforts have elucidated pathophysiological processes that also reflect the illness’ heterogeneity, there are no biomarkers used in clinical practice to aid diagnosis or to guide treatment. This may be due to the scale of the challenge. Scalable and accurate study design, alongside representative, replicable and robust testing of accurate biomarkers is necessary to advance understanding of the complexity of psychosis and its clinical application.^6^

Given the need for scale and replication, blood, plasma and serum may offer accessible samples for biomarker identification, with potential to improve clinical care for people who experience psychosis and to better target interventional studies and drug trials.^6^ The Severe Mental Illness Longitudinal Evaluation (SMILE) BioResource is designed to address the gap in long-term storage of biological samples and phenotypic data to support future discovery science involving people with psychosis.

A bioresource is a collection of biological samples and clinical data from consented individuals with possibilities for follow-up contact. ‘Biorepository’ and ‘biobank’ are other names used to describe a facility that receives, processes, stores and manages access to biological samples.^7^ The primary aims of a bioresource are to archive and distribute collected samples and data to support scientific research.^7^ Collected samples are quality-controlled and can be processed for serum or plasma, frozen, and stored for genomic, proteomic, metabolomic, inflammatory and other testing. Samples can then be requested for research by biomarker or phenotype.^8^ Quality control and standard operating procedures for sample collection and handling are essential across large, multisite resources to minimise avoidable heterogeneity and confounds.

The SMILE BioResource is funded by the National Institute for Health and Care Research (NIHR) Mental Health Translational Research Collaboration Mission; it is a collaborative project involving researchers at the University of Oxford, the University of Birmingham, Cardiff University, King’s College London and the national NIHR BioResource. The SMILE BioResource will collect relevant information for understanding how biological (eg, genomic, proteomic, inflammatory) and environmental factors influence psychosis presentation, symptom profile, prognosis and treatment response. The SMILE BioResource will provide a recontactable cohort with stored biosamples for recall to future studies, including trials and observational studies. A bioresource with extensive mental health data and biosamples offers significant potential for research into psychosis for many years to come.

## Methods and analysis

### Design

All collected data and samples will be transferred to the NIHR BioResource for long-term storage by the end of January 2028. The NIHR BioResource is a research tissue bank that provides researchers with access to a large panel of consented volunteers, along with their genetic, health and lifestyle data and stored samples. The NIHR BioResource comprises a collection of over 300 000 recallable volunteers, including individuals with rare and common illnesses as well as individuals from the general population. The unique contribution of the SMILE BioResource will be a focus specifically on people with psychosis to ensure they are represented within this established and robust resource.

Beginning recruitment in November 2025, the SMILE BioResource is funded to recruit 2000 individuals with a recorded diagnosis that meets criteria for psychosis as defined by ICD-11, including 6A20 (schizophrenia), 6A21 (schizoaffective disorder), 6A22 (schizotypal disorder), 6A23 (acute and transient psychotic disorder), 6A24 (delusional disorder), 6A25 (symptomatic manifestations of primary psychotic disorders), 6A2Y (other specified primary psychotic disorder) and 6A2Z (schizophrenia or other primary psychotic disorders, unspecified).

While some definitions of SMI also include bipolar disorder and major depressive disorder, these cohorts are being collected as part of the Genetic Links to Anxiety and Depression NIHR Bioresource study. SMILE will focus on SMI within the scope of psychosis. UK primary care data reported an incidence of 9.2 per 100 000 for schizophrenia and 22.3 per 100 000 person years for other psychotic disorders compared with 15.0 per 100 000 person years for bipolar disorder.^9^ This indicates that within incident cases of psychosis, schizophrenia and non-schizophrenia psychoses make up the largest proportion of potential SMI diagnoses currently underserved within national Bioresource infrastructure.

#### Informed consent

Informed consent will be obtained prior to any research procedures. To join the SMILE BioResource, patients must consent to:

- Provide a biological sample and data at the study visit.
- ‘Access in principle’ to their electronic health records and other relevant records.
- Their data and biosample being stored long-term by the NIHR BioResource.
- Their de-identified data being used for research approved by the NIHR BioResource.
- Be invited to future research for which they may be eligible.

Where study sites have trials or cohort studies also recruiting participants with SMI, these participants may also be presented with information about co-consenting to the SMILE BioResource.

People with SMI may have difficulty with insight, memory and cognition which makes research participation more challenging. However, capacity to consent to research, as with any capacity decision, is context dependent and specific to a particular question or setting. Participants may have positive symptoms but may retain capacity to make a decision about study participation. All research staff who recruit to the SMILE BioResource are trained in good clinical practice and work with clinical teams to ensure that support is in place for communicating study information to potential participants. Some patients may be too unwell to provide consent to participate in SMILE, for example during an acute episode; these patients can instead be approached later in their illness course once they have recovered.

Based on population descriptives, we estimate there will be ∼5% of patients with severe, intractable treatment resistance that affects capacity to join research longer term.^10^ We will monitor recruitment figures, Positive and Negative Syndrome Scale (PANSS) and Clinical Global Impression-Schizophrenia (CGI-S) scores, and we will assess our data against representative cohorts with the most severe illness scores from published data. We will consider adding a modification to include consultee opinion in keeping with Health Research Authority guidance.

### The study visit

Each study visit will last between 30 minutes and 60 minutes and will be conducted by a trained clinical researcher. Participants will be able to bring a close relative, friend, care coordinator or peer support worker to the study visit. Participants will be offered a £25 shopping voucher to thank them for their participation.

### Research questions

The first stage of the SMILE BioResource will involve the collection of biosamples and data at study sites as well as analysis by SMILE study investigators. Our initial primary research aim are:

- To establish the prevalence of cognitive difficulties and physical comorbidity and their association with length of illness and sociodemographic factors.

Multivariate methods will be employed to characterise association between independent variables (eg, length of illness, sociodemographic factors) and dependent variables (eg, cognitive performance, physical health symptoms).

- To establish the influence of biological risk factors, for example, genomic risk on time and severity of physical health comorbidity and cognitive decline.

Similarly to our first objective, a multivariate approach will be employed to characterise the association between biological risk factors (eg, robust polygenic risk scores) and (1) cognitive (2) physical health outcomes.

The second stage of the SMILE BioResource will involve long-term storage of all study data and biosamples as well as the opportunity for academic and industry researchers to apply to the NIHR BioResource to use these data and biosamples for research and/or to invite SMILE BioResource participants to future research.

### Patient and public involvement engagement and participation

The SMILE BioResource involves a group of patient and public contributors from England and Wales. This group comprises people with SMI and experience of caring for people with SMI. Patient and public involvement engagement and participation (PPIEP) ensures the SMILE BioResource reflects the needs of those with SMI, and that language used is accessible and appropriate. This group co-designed aspects of the study with the SMILE BioResource research team including patient-facing documents and study branding. Two members of this PPIEP group contribute to quarterly study steering committee meetings.

#### Participant selection

People diagnosed with psychosis in the UK receive care in secondary mental health services, which offer pharmacological and/or psychological treatment, as well as community and vocational support. When presenting with first-episode psychosis, Early Intervention in Psychosis services are offered for the first 3 years in England and parts of Wales. After 3 years, the care needs of people with SMI are provided by community mental health services and primary care. In other parts of the UK, care is delivered to people with first-episode psychosis by community mental health teams.

Secondary care NHS caseload information (or paper records) will be screened for potential participants who have a recorded diagnosis of psychosis by research nurses/clinical researchers embedded in services, as per individual site processes.

Potential participants will be approached by research or clinical staff at a convenient time and place, including clinic appointments, physical health checks or home visits. Participants must be able to give informed consent, they must be aged 16 years or older, and their medical records must contain a history of psychosis that meets ICD-11 criteria as above. Potential participants are free to decline participation or to withdraw at any time for any reason without prejudice to future care, without affecting their legal rights and with no obligation to give a reason for declining/ withdrawal.

### Outcome measures

1. Biosample (blood or saliva sample).
  Up to 50 mL of blood may be taken from a participant at one point in time only. Blood samples will be collected using blood collection tubes (eg, vacutainer; 2× EDTA and 1× serum tube). Where possible, blood taking is synchronised to the morning and when samples are being taken for standard clinical care. Blood samples must be sent on the same day as collected to the UK Biocentre (where the NIHR BioResource stores research samples). Extracted DNA will be stored in −80°c freezers with careful temperature logging and monitoring.
  Self-collection saliva kits will be used to collect saliva samples if it is not possible to collect blood. DNA from saliva can be used for most genetic analysis but not for whole-genome sequencing.
2. Patient-reported outcome measures (PROMs).
  PROMs will be collected to capture a patient’s experiences and perspectives on their health and treatment. PROMs include the Altman self-rating mania scale^11^; the Bespoke Adapted Chemical Use, Abuse and Dependence Scale for Alcohol and Drug Use^12^; a Child Trauma Screen^13^; a Generalised Anxiety Disorder Assessment^14^; the Patient Health Questionnaire^15^ and a Health and Lifestyle Questionnaire developed by the NIHR BioResource.
3. Clinician-reported outcome measures (CROMs).
  CROMs will be completed by a trained clinical researcher. They are important to assess and report on a patient’s health status, illness severity and functional abilities. The CROMs that we will use include the CGI-S scale^16^; the PANSS 6-item^17^; and the functional remission of general schizophrenia.^18^
  Clinical data, including vital signs (systolic and diastolic blood pressure and pulse rate); full blood count and differential, biochemistry, lipids and glucose; C-reactive protein; ECG; regular medication; and physical health diagnoses will all be obtained from patient health records.
4. Demographic information.
  Date of birth, sex at birth and gender, area postcode for deprivation indices and ethnicity, as recorded by the 2021 UK census ethnicity classification.
5. Cognition.
  The digital Symbol-Symbol Substitution Task (SSST) is an alternative version of the digit-symbol substitution task (DSST), which is a well-established psychometric test paradigm used to measure various aspects of cognition, including psychomotor speed, attention and visuospatial function.^19^ The SSST shows high correlation with the standard DSST (r=0.8) and is being used in multiple patient populations, including those with schizophrenia and other psychotic disorders.^20^,^22^ For the SSST, participants are asked to match a symbol to another symbol according to pre-existing pairs. We will collect data for the SSST using a computing device (eg, laptop, tablet) that will record the number of correct and incorrect responses within 90 seconds.
6. Relevant information from care notes including recent or current hospitalisation, home treatment episodes, medication prescription (including current drug, dosing, frequency and compliance) and recent changes, and new mental health or physical health diagnoses.
7. Permission to access the participant’s electronic health records and other records (‘access in principle’) that may be relevant for research.
  ‘Access in principle’ means that for future studies, only records that are strictly relevant for approved research purposes will be accessed. At the point of data collection, only medical records that are strictly relevant to the study of SMI are collected as CROMs, for example, information about regular medications and blood results.

### Stage 2: data analysis at the NIHR BioResource

Biosamples collected for SMILE will be processed and stored in the UK Biocentre (Milton Keynes, UK). Blood samples will be centrifuged to isolate serum or plasma. Aliquots of whole blood, serum and plasma will then be stored at –80 degrees Celsius to preserve the long-term integrity of important biological molecules, such as DNA, proteins and other metabolites, for downstream processing. Saliva samples can be stored at ambient room temperature for several months prior to processing. Processing and storage of biosamples is described in detail by the NIHR BioResource.

Following collection and processing of SMILE BioResource biosamples, it will be possible for academic and industry researchers to apply to the NIHR SMILE BioResource to conduct further research. For example, genomic sequencing can be applied to both saliva-based and blood-based samples to identify variation in DNA sequences with relevance to disease states in psychosis. Similarly, proteomic and metabolic sequencing may help to identify abnormal proteins and metabolites which are important to the pathogenesis of SMI. As well as the identification of known molecules, unbiased and discovery-driven sequencing technologies, such as mass spectrometry, may facilitate the identification of novel biomarkers for diagnostic and/or therapeutic purposes. Finally, whole blood may undergo further processing to isolate peripheral mononuclear blood cells for use in functional assays, stem cell models and single cell sequencing studies.

Biological data accessible to researchers can be analysed in conjunction with linked environmental and clinical data, including smoking status, patient symptomatology, blood biochemistry, medication exposure and other basic clinical investigations. Access to these large and related datasets may facilitate robust descriptive and analytical epidemiological studies into SMI, as well as genetic and integrative multiomic approaches to biomarker discovery and risk prediction. Statistical techniques, such as mendelian randomisation analyses, may also be applied to these data to infer causal relationships between exposures and healthcare outcomes.

## Ethics and dissemination

The SMILE BioResource study is sponsored by the University of Oxford and received full ethical approval from Wales REC 2 in July 2025 (REC Reference 25/WA/0202; IRAS number: 350558). The objectives of the SMILE BioResource study received extensive peer review as part of the award for the NIHR Mental Health Translational Research Collaboration Mission.

The NIHR BioResource is funded by the NIHR. The Research Tissue Bank has been reviewed and given a favourable opinion by the East of England—Cambridge Central Research Ethics Committee (REC Reference 22/EE/0230; IRAS number: 313104). Cambridge University Hospitals NHS Foundation Trust is responsible for the management of the NIHR BioResource Research Tissue Bank.

### Safeguarding

The SMILE BioResource research team has a duty of care to clearly explain the study purpose, what participation will involve, the voluntary nature of the study and withdrawal options. Some of the study questionnaires include sensitive questions, for example, about childhood trauma, and so participants and the research team are reminded that they should only proceed with certain questions if the participant feels comfortable to do so.

The participant will be signposted to relevant support services, should they feel they may need them. If, as part of study assessments, the participant discloses a risk to themselves or others, the study team has an obligation to pass this on to the care provider/mental health team of the participant, if this information is not already known.

### Data management

SMILE BioResource data will be collected, stored and transferred to the NIHR BioResource, via Global Initiative (ISO 27001 & 9001; CyberEssentials; NHS DPST certified) on behalf of the project management office which is based at the University of Oxford. Study data will be collected using a digital platform called Trial Deck (TD). TD is a secure, web-based research platform designed to support the conduct of clinical studies, providing (1) participant enrolment, e-consent and eligibility screening; (2) electronic case report form and questionnaire capture, including patient-reported outcomes and e-diary modules; (3) audit trails for participant engagement and researcher activity; and (4) integrations for external data sources such as laboratories, wearables and electronic health records.

**Figure 1:**
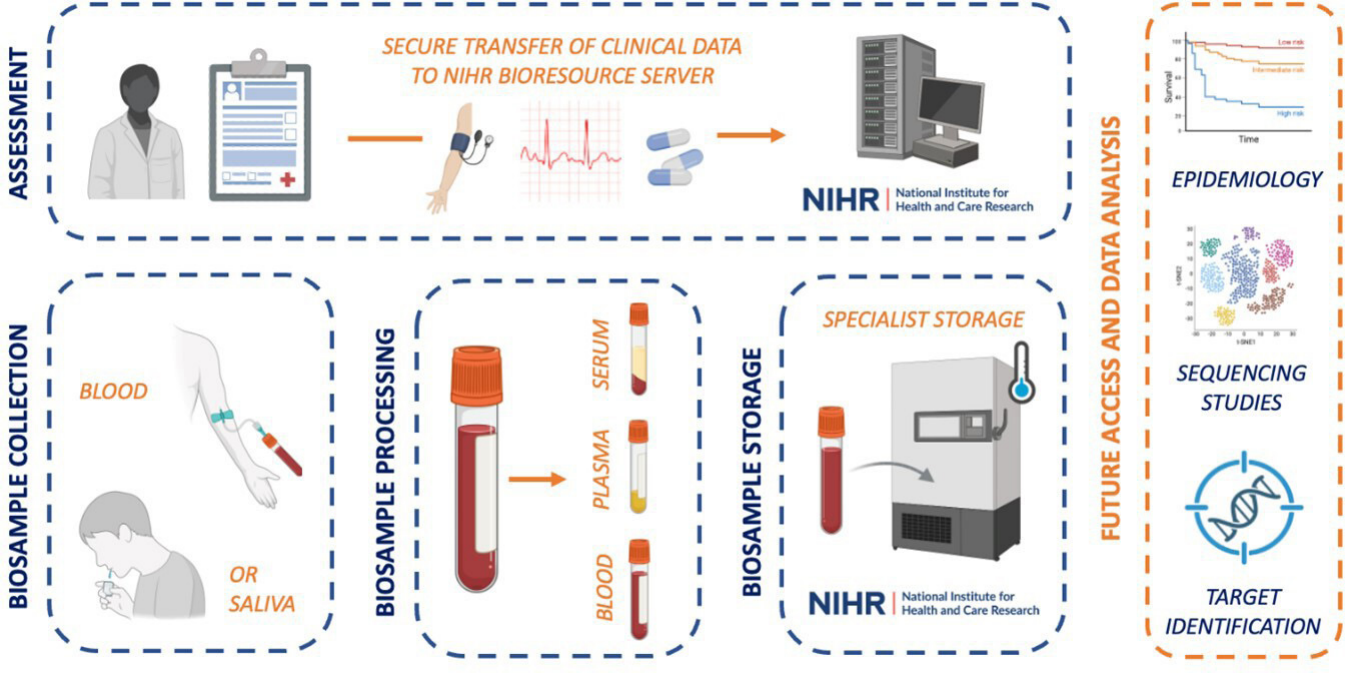
Data flow through the SMILE BioResource. Overview of the collection, processing and storage of participant data and biological samples, from acquisition at the study visit through processing, to long-term storage within the NIHR BioResource. SMILE, Severe Mental Illness Longitudinal Evaluation; tSNE, t-distributed stochastic neighbor embedding.

Study data will be transferred from the TD platform to the NIHR BioResource repository on an agreed schedule. After biosamples have been collected at study sites, they will be sent directly to the UK Biocentre, which is where the NIHR BioResource stores its samples. Figure 1 provides an overview of sample and data flow for the SMILE BioResource.

### Data governance

The SMILE BioResource study complies with the UK General Data Protection Regulation (UK GDPR) and the Data Protection Act 2018, which stipulates pseudonymisation as a safeguard for processing personal data. Identifying details will be held separately from other study data and participants will be assigned a unique ID that is linkable to study data that they provide. The link between personal data and study ID numbers will be maintained in a safe computing environment by the NIHR BioResource and only accessible to authorised personnel. A Data Protection Impact Assessment for the study details how appropriate safeguards are in place for processing personal data.

Release of samples and/or data will be covered by a Material/Data Transfer Agreement (MTA/DTA) with all individuals/organisations requesting access, whether they reside within or outside the UK. The MTA/DTA is a legally binding document that regulates the use of samples and data to ensure that standards are maintained. Where samples are released and there is a surplus after the study is completed, the return, disposal or storage will be described in the study specific protocol and research ethics application.

### Data sharing

The NIHR BioResource considers requests for de-identified samples following an application and review process. Applications to access study samples or study data are made through the NIHR BioResource website (see https://www.bioresource.nihr.ac.uk/using-our-bioresource/apply-for-samples/). Applicants are required to explain how they will use the samples/data that they request. Applications are reviewed by a NIHR BioResource committee. This is referred to as ‘managed access’. Requests for samples/data could come from researchers, from the public and charitable sector, or from commercial and pharmaceutical companies.

Applicants seeking access to data and/or samples held by the NIHR BioResource may be based in the UK or internationally. Successful applicants will be obliged to keep data safe and secure by accepting the terms of a data transfer/data access agreement. Where data are shared outside the UK, organisations must have appropriate security measures to protect study data. These measures must be consistent with data security obligations in the UK.

### Participant recontact

The NIHR BioResource will manage any recontact with SMILE BioResource participants for the purpose of inviting them to take part in further research studies, including clinical trials, or to donate fresh samples for additional analysis. Recontact is currently available and scheduled until current funding expires in January 2038. However, our vision is one of a long-term and growing resource.

Follow-up studies may involve clinic visits, telephone assessments or home visits by study staff. The maximum number of invitations to studies involving face-to-face recall is approved to be four in every 12-month period. Within this same 12-month period, participants may be invited to up to four further online-only studies (eg, questionnaires or cognitive test-based studies) that they can complete in their own time.

## Discussion

To date, there has been a paucity of well-characterised, openly available resources for targeted biomarker investigation involving individuals with psychosis. Such resources are needed given that scientific discovery, assay development and technological advances are occurring at pace. It is likely that current treatment efficacy is diminished by an inability to stratify patients into subtypes based on biomarkers (such as genomic, inflammatory or metabolic subtypes) and to deliver tailored treatment pathways that address individual disease processes. Furthermore, reliance on cross-sectional data has limited investigation into biomarkers and their relation to disease trajectory. The implementation of screening programmes and timely preventative interventions remains a challenge.

The SMILE BioResource will offer an invaluable resource, developed with patients, to help address these unmet needs. Through longitudinal, in-depth phenotyping of a large patient cohort with a history of SMI and psychotic disorder, together with stored samples, the SMILE BioResource will provide a wealth of clinical and biological data that reflects the heterogenous and dynamic nature of this mental illness. The SMILE BioResource could help to advance understanding of the relationship between biological and environmental risk factors and their influence on psychosis presentation, symptom profiles, prognosis and response to treatment.

By aligning with the core missions of the NIHR Mental Health Translational Research Collaboration and the NIHR Bioresource, SMILE will provide a key substrate for multiomics and next-generation sequencing technologies, enabling investigation into the underlying biology of psychosis. Ultimately, the SMILE BioResource will facilitate early-stage translational research into novel drug targets and innovative therapies to improve clinical practice and mental health through discovery medicine.

## References

[1] Naylor C, Parsonage M, McDaid D (2012). Long-term conditions and mental health: The cost of co-morbidities.

[2] Public Health England (2018). Severe mental illness (smi) and physical health inequalities: briefing. https://www.gov.uk/government/publications/severe-mental-illness-smi-physical-health-inequalities/severe-mental-illness-and-physical-health-inequalities-briefing/.

[3] Rawani NS, Chan AW, Dursun SM (2024). The Underlying Neurobiological Mechanisms of Psychosis: Focus on Neurotransmission Dysregulation, Neuroinflammation, Oxidative Stress, and Mitochondrial Dysfunction. Antioxidants (Basel).

[4] Tost H, Alam T, Meyer-Lindenberg A (2010). Dopamine and psychosis: theory, pathomechanisms and intermediate phenotypes. Neurosci Biobehav Rev.

[5] Owen MJ, Sawa A, Mortensen PB (2016). Schizophrenia. Lancet.

[6] Comes AL, Papiol S, Mueller T (2018). Proteomics for blood biomarker exploration of severe mental illness: pitfalls of the past and potential for the future. Transl Psychiatry.

[7] Grizzle WE, Bledsoe MJ, Al Diffalha S (2019). The Utilization of Biospecimens: Impact of the Choice of Biobanking Model. Biopreserv Biobank.

[8] NIHR BioResource (2025). Apply for samples. https://www.bioresource.nihr.ac.uk/using-our-bioresource/apply-for-samples/.

[9] Hardoon S, Hayes JF, Blackburn R (2013). Recording of severe mental illness in United Kingdom primary care, 2000-2010. PLoS One.

[10] Hostiuc S, Rusu MC, Negoi I (2018). Testing decision-making competency of schizophrenia participants in clinical trials. A meta-analysis and meta-regression. BMC Psychiatry.

[11] Altman EG, Hedeker D, Peterson JL (1997). Altman Self-Rating Mania Scale (ASRM). APA PsycTests.

[12] McGovern MP, Morrison DH (1992). The Chemical Use, Abuse, and Dependence Scale (CUAD). Rationale, reliability, and validity. J Subst Abuse Treat.

[13] Lang JM, Connell CM (2017). Child Trauma Screen (CTS). APA PsycTests.

[14] Spitzer RL, Kroenke K, Williams JBW (2006). A brief measure for assessing generalized anxiety disorder: the GAD-7. Arch Intern Med.

[15] Kroenke K, Spitzer RL, Williams JBW (2001). The PHQ-9: validity of a brief depression severity measure. J Gen Intern Med.

[16] Haro JM, Kamath SA, Ochoa S (2003). The Clinical Global Impression-Schizophrenia scale: a simple instrument to measure the diversity of symptoms present in schizophrenia. Acta Psychiatr Scand Suppl.

[17] Østergaard SD, Lemming OM, Mors O (2016). PANSS-6: a brief rating scale for the measurement of severity in schizophrenia. Acta Psychiatr Scand.

[18] Llorca P-M, Lançon C, Lancrenon S (2009). The “Functional Remission of General Schizophrenia” (FROGS) scale: development and validation of a new questionnaire. Schizophr Res.

[19] Jaeger J (2018). Digit Symbol Substitution Test: The Case for Sensitivity Over Specificity in Neuropsychological Testing. J Clin Psychopharmacol.

[20] Heinrichs RW, Ruttan L, Zakzanis KK (1997). Parsing schizophrenia with neurocognitive tests: evidence of stability and validity. Brain Cogn.

[21] Bachman P, Reichenberg A, Rice P (2010). Deconstructing processing speed deficits in schizophrenia: application of a parametric digit symbol coding test. Schizophr Res.

[22] Amaresha AC, Danivas V, Shivakumar V (2014). Clinical correlates of parametric digit-symbol substitution test in schizophrenia. Asian J Psychiatr.

[23] Rugkåsa J, Canvin K (2011). Researching mental health in minority ethnic communities: reflections on recruitment. Qual Health Res.

