# Supplementary material for: Severe Mental Illness Longitudinal Evaluation (SMILE): protocol for establishing a cohort and bioresource for UK-based patients with psychosis
Source: BMJ Open. 2026 Jul 1;16(7):e116077. doi: 10.1136/bmjopen-2025-116077 (PMC13331059; doi:10.1136/bmjopen-2025-116077)
Supplement: Supplementary file 1 [file bmjopen-2025-116077.reviewer_comments.pdf]

PEER REVIEW HISTORY

BMJ Open publishes all reviews undertaken for accepted manuscripts. Reviewers are asked to complete a checklist review form and are provided with free text boxes to elaborate on their assessment. These free text comments are reproduced below.

ARTICLE DETAILS

Title (Provisional)

Severe Mental Illness Longitudinal Evaluation (SMILE): protocol for establishing a cohort and bioresource for UK-based patients with psychosis

Authors

Quinn, Anthony; Johnson, Thomas; Bieles, Julie; Booth, Lesley; McCutcheon, Rob; Bradley , John; Kingston, Nathalie; Breen, Gerome; Harshfield, Amelia; Murray, Graham K.; Walters, James; Perry, Benjamin I.; Ul Haq Katshu, Mohammad Zia; Kendall, Kimberley; Upthegrove, Rachel

VERSION 1 - REVIEW

**Reviewer**	**1**
**Name**	**Fachiroh, Jajah**
**Affiliation**	**Universitas Gadjah Mada, Histoogy and Cell Biology**
**Date**	**02-Mar-2026**
**COI**	**None**

"The NIHR SMILE BioResource: protocol and research resource" by Quinn et al., describe protocol for biobanking of SMI and related conditions, that provide a global extensive research source for clinical and epidemiology as well as biomarkers development studies.

Several questions related to the protocol are listed below:

1. Authors plan to include 2,500 participants. What is the rationale of the numbers, and according to the registry what is the proportion of participants numbers related to different types of SMI (ICD-11), and will it be adequately answer the multifactorial questions?

2. Inclusion criteria needs direct agreement of the participants. Patients with severe mental illness may lack the decisional capacity to provide informed consent due to impaired mental status. How big is the fraction of this condition expected, and will it interfere with the proposed number of participants?

3. How does "access in principle" applied in data collection, in particular whether all clinical data be collected (from medical record or solely through the study) or a path is made in

order for studies (through the biosource) to inquire for data to be accessed from medical record/ biosource database?

4. Sample collection:

a. Please add further explanation on how 50ml of peripheral blood will be collected? will it use different types of anticoagulant, no anti coagulant, and analytes planned

I believe that is included for PBMC collection as well, as it has to be done prior to final sample storage in -80C.

VERSION 1 - AUTHOR RESPONSE

Reviewer: 1

Comments to the Authors:

Several questions related to the protocol are listed below:

1. Authors plan to include 2,500 participants. What is the rationale of the numbers, and according to the registry what is the proportion of participants numbers related to different types of SMI (ICD-11), and will it be adequately answer the multifactorial questions?

Thank you for your feedback. We have now better explained the different types of SMI. We have re-drafted the Introduction section (see page 3) to better clarify from the outset our use of SMI, and Psychosis. We have moved ICD-10 codes into the Methods section for clarity (see bottom of page 4).

We have secured funding for up to 2500 participants but aim for SMILE to be a growing resource and highlight that this will provide illness specific samples and data to the wider 300,000 NIHR BioResource across other disease areas.

2. Inclusion criteria needs direct agreement of the participants. Patients with severe mental illness may lack the decisional capacity to provide informed consent due to impaired mental status. How big is the fraction of this condition expected, and will it interfere with the proposed number of participants?

This is an important point about capacity to consent. Within the Informed Consent section (see page 5), we have now included further information about capacity to consent and estimations for the proportion of potential participants that this will likely affect.

3. How does "access in principle" applied in data collection, in particular whether all clinical data be collected (from medical record or solely through the study) or a path is made in order for studies (through the biosource) to inquire for data to be accessed from medical record/ biosource database?

We have now better explained what “access in principle” means within item 7 of our Outcome Measures section (see page 9).

4. Sample collection:

a. Please add further explanation on how 50ml of peripheral blood will be collected? will it use different types of anticoagulant, no anti coagulant, and analytes planned

I believe that is included for PBMC collection as well, as it has to be done prior to final sample storage in -80C.

We have now added further details about SMILE BioResource sample collection procedures within item 1 of our Outcome Measures section (see bottom of page 7).
